# Miscellany

**Published:** 1890-09

**Authors:** 


### Miscellany.

### RECENT PATENTS IN MEDICINE AND SURGERY.

Reported by CHARLES J. GOOCH, Esq., Patent Attorney, Washington, D. C.

AIROZONIZING apparatus, J. C. Kennedy, Detroit, Mich.; making Chlorine, E. Solvay, Brussels, Belgium ; Evaporating apparatus, T. Guant, Brooklyn, N. Y.; Nursing bottle nipple, (two patents,) A. C. Eggers, Brooklyn, N. Y.; Speculum, R. P. & C. H. McCully, Brooklyn, N. Y.; Reservoir syringe, S. E. Cook, Holdrege, Neb.; Temperature regulator, L. F. Easton, La Crosse, Wis.; Capsule dipping machine, J. Krehbiel, Kalamazoo, Mich.; Capsule stripping machine, J. Krehbiel, Kalamazoo, Mich.; Cutting gelatine capsules, J. Krehbiel, Kalamazoo, Mich.; Dental engine motive gear, P. Brown, Montreal, Can.; Artificial denture, N. J. Goodwin, Hartford, Conn.; Disinfectant, H. M. Baker, Brooklyn, N. Y.; Surgical forceps, F. W. Groth, Chicago, Ills.; Administering medicine to animals, W. H. H. Doty, Paterson, N. J.; Obtaining acetic acid and methyl alcohol, F. C. Alkier, Wieselburg-on-the-Erlauf, Austria, Germany ; Rosinduline sulpho acid, C. S. Chraube, Ludwigshafen-on-the-Rhine, Germany ; Remedial cosmetic, C. J. Wilkins, Denver, Col.; Fountain syringe, C. A. Tatum, New York, N. Y.; Soda water dispensing apparatus, J. W. Tufts and J. Ramey, Medford, Mass.; Making thio-oxydiphenylamine, M. Lange, Amersfoot, Neth.

July 22 : Making acetic acid, I. A. F. Bang and M. C. A. Ruffin, Paris, France ; Producing bisulphites, C. Cornwell, Ypsilanti, Mich.; Bisulphite solutions, T. P. Burgess, Dedham, Mass.; Dental mold, L. F. Seeger, Jr., Ahnapee, Wis.; Fracture apparatus, T. M. Miller, Medford, Wis.; Inhaler, J. D. Averell, Manchester, N. J.; Inhaler and respirator, H. F. Williams, Brooklyn, N. Y.; Obtaining oxygen from air, A. Brin, London, England ; Poultice holder, C. S. Hirst, Philadelphia, Pa.; Surgical apparatus, W. L. Hendrick, Boston, Mass.; Surgical knife, G. Dittert, Neustadt, Saxony ; Surgical splint, L. Reenstierna, Boston, Mass.; Clinical thermometer case, H. J. Green, Brooklyn, N. Y.; Artificial tooth, C. H. Land, Detroit, Mich.

Patents granted July 29, 1890 : Abdominal supporter, H. E. Rogers, Portland, Me.; Dental Vulcanizer, J. E. Quinn, Boston, Mass.; Inhaler, G. W. Nutz, Philadelphia, Pa.; Inhaler and Spirometer, W. A. Shepard, Elgin, Ill.; Artificial leg, G. Beacock,

Montreal, Canada ; Pharmaceutical powder divider, W. F. Dessau, Chicago, Ill.; Obtaining pepsin, J. Brill, Philadelphia, Pa.; Salve, J. J. Ryan, Brooklyn, N. Y.; Surgical splint, G. Beacock, Montreal, Canada.

Patents granted Aug. 5, 1890 : Dental mallet and pliers, A. O. Corey, Council Grove, Kan.; Disinfectant, W. F. Simes, Philadelphia, Pa.; Inhaler, J. S. Kinnear, Golden, Colo.; Artificial leg, H. C. Wintermute, Kansas City, Mo.; Artificial limb, H. S. Swank, Columbus, O.; Solidified perfume, G. H. Dubelle, New York, N. Y.; Washing the fumes of sulphur, Montgomery & Warnke, New Orleans, La.

Atomizer, P. J. McElroy, Cambridge, Mass.; Hospital bedstead, F. L. Bryant, Chicago, Ill.; Capsule-dryer, T. C. Merz, Detroit, Mich.; Nitro-cellulose, G. M. Mowbray, North Adams, Mass.; Spray diffuser, E. W. Mackensie-Hughes, Chicago, Ill.; Truss, Brownlow & Warner, Ogdensburg, N. Y.

### TRADE-MARKS.

Cough remedies and blood-purifying compounds, F. V. D. Smith, Croton Falls, N. Y.; Deodorizers, disinfectants, liniments, ointments, Ellingwood & Co., Lowell, Mass.; Injections and lotions for venereal diseases, W. M. Ryals, Lumber City, Ga.; Medicine for headache, nervousness, sea-sickness and nausea, H. deWindt, London, England ; Tonic and blood purifier, Kopp & Litchenberger, Asheville, N. C.; Tonic and remedy for asthma, &c., Kopp & Litchenberger, Asheville, N. C.; Remedy for liver and kidney complaint, O. S. Greenleaf, Holyoke, Mass.; Remedy for diseases of the brain, nerves and muscles, Peruvian Medicine Co., Cincinnati, O.; Remedy for diseases of the nervous and sexual system, C. S. Hardy, Boston, Mass.

Trade-marks issued July 22, 1890 : Artificial milk, A. Schnell, St. Louis, Mo., the word, "Kefyr ;" Tonics and malt extracts, Broening Bros., Lincoln, Neb., a star in outline, with a circle upon the center of the star ; Syrup of amorphous quinine, Paris Medicine Co., Paris, France, the word "Febriline ;" Medicinal tablets, Sharp & Dohme, Baltimore, Md., the word "Pan-Peptic ;" Liquid ergot of rye, Sharp & Dohme, Baltimore, Md., the word "Ergotole ;" Liniment, C. Schlayer, Philadelphia, Pa., the words "Pain Slayer ;" Vegetable compound for certain diseases, A. Swanson, Jamestown, N. Y., the word "Kalmar ;" Bitters, R. Y. McWilliams, New Orleans, La., the word "Beacon ;" Carbonated beverages,

Union Bottling Co., New York, N. Y., the words "The Golden Key Brand" and the representation of a key; Flavoring syrup for soda water, H. B. Mykrantz, Kansas City, Mo., the words "Champagne Mist;" Perfumery, J. L. Grossmith, London, England, the word "Hasu-no-hana;" Compounded medicines, The Home Medication Co., New York, N. Y., heraldic garter, words "The Home Medication Co.;" representation of a mortar and pestle, a flying dove, and the words "Health," "Purity," "Beauty;" Pills, The Aphro Medicine Co., Portland, Ore., the words "Faber's Golden Female;" Remedy for dyspepsia, catarrh of the stomach, ulcers and other microbian diseases, The Drevet Mfg. Co., New York, N. Y., the word "Hydrozone."

Trade-marks issued July 29, 1890: Medicated toothpicks, E. E. Burson, Rockford, Ill., representation of a rustic diamond-shaped frame formed of toothpicks, enclosing the words "Medicated Toothpicks;" Remedy for diseases of the digestive organs, F. N. Glass, New York, N. Y., the word "Tryphosa;" Certain named medical remedies, W. G. Lewi, Albany, N. Y., a portrait of the face of Dr. John Swinburne, formerly of Albany, N. Y.; Medicines for certain diseases of men and animals, E. S. Sloan, Boston, Mass., a portrait of the registrant; Chemical preparation for bleaching purposes, The Drevet Mfg. Co., New York, N. Y., the word "Ozonine;" Remedy for the destruction of bacteria, microbes and germs in the human system, Drevet Mfg. Co., New York, N. Y., the word "Glycozone;" Florida water, American Florida Water Co., New York, N. Y., representation of a vase containing a white, a red, and a yellow rose; Remedy for wounds, Sommer Bros., Hawkinsville, Ga., a circular band, at opposite sides of which are arranged two crescent-shaped figures.

Trade-marks issued Aug. 5, 1890; Emollient, W. Casler, Chicago, Ill., the word "Oriental;" Ointment, W. B. Eddy & Co., Whitehall, N. Y., the words "Quinn's Ointment;" Peroxide of hydrogen, Drevet Mfg. Co., New York, N. Y., the word "Marchand."

Trade-marks issued Aug. 12, 1890: Rheumatic liniment, Smith & Parr, Washington, D. C., the word "Rheumaticide" in red letters and in a curved line; Remedies for diseases of the throat and lungs, T. V. Sords, Cleveland, O., the word "Broncha" in script letters; Oil of lemon, Dodge & Olcott, New York, N. Y., the word "Limoniano;" Oil of bergamot, Dodge & Olcott, New York, N. Y., the word "Bergamottino;" Oil of orange, Dodge & Olcott, New York, N. Y., the word "Aranciano;" Blackberry brandy,

Rheinstrom Bros., Cincinnati, O., a monogram of the registrant's firm, "R. Bros.," surrounded by a wreath composed of a blackberry vine; Remedy for certain named diseases, S. E. Root, Rochester, N. H., the words, "Bureau of American Remedies."

LABELS.

Labels registered for the past month: Dr. W. T. Rowsey's Never-Failing Whooping Cough Remedy, H. F. Rowsey, Toledo, O.; Syrup of Figs, California Fig Syrup Co., San Francisco, Cal.; Mrs. L. Schmidt & Co.'s Healing Liniment, Mrs. L. Schmidt & Co., New York; Almond Nut Salve and Almond Nut Cream, M. E. Murray, Chicago, Ill.; Hot Springs, Eczema Powder, N. Lichty, Des Moines, Iowa; Half Dollar Box of Chemicals, H. L. Corbit, Brooklyn, N. Y.; Medicated Food, J. Elliott, Indianapolis, Ind.; Schreibers' Tonic, J. D. Schreiber, Allentown, Pa.; Glycozone, for a medical remedy, Drevet Mfg. Co., New York, N. Y.; Syrup of Figs, California Fig Syrup Co., San Francisco, Cal.; Casey's, for an adhesive plaster, F. M. Casey, Mount Vernon, N. Y.

---

THE volume descriptive of the Johns Hopkins Hospital, prepared by Dr. John S. Billings at the request of the Trustees, is now ready for distribution. It contains fifty-six large quarto plates, phototypes and lithographs, with views, plans and detail drawings of all the buildings, and the interior arrangement—also wood-cuts of apparatus and fixtures. It has 116 pages of letter-press, describing the plans followed in the construction, and giving full details of heating apparatus, ventilation, sewerage and plumbing.

The volume is issued in three styles: bound in half-morocco, $9.00; bound in cloth, $7.50; bound in light boards, $5.00. In the copies bound in half-morocco and cloth the plates are separately mounted on guards. The edition is limited to 750 copies.

The volume will be sent, expressage paid by the publisher, on receipt of the sum named above.

Orders and subscriptions should be addressed to THE JOHNS HOPKINS PRESS, BALTIMORE, MARYLAND.

---

"THERE'S MILLIONS IN IT."—The neatness with which German drug houses patent and trade-mark a medicine, and then make the doctors free advertisers for it, betokens a certain shrewdness in the German mind and vacuity in the American mind that is not credited to them respectively by tradition. When an American patented and trade-marked medicine goes to Europe, it is quietly led to the door and the official boot-toe makes one vigorous outward sweep, there is a dull thud and — exit patent medicine. Antipyrin, sulphonal, and other German patent medicines meet with quite a different reception in America, as everyone knows, though perhaps everyone does not know that they are virtually patent medicines. Mrs. Winslow, Castoria, S. S. S., H. H. Warner, and other great medicine men of Yankeedom, ought to go to Europe and sit at the feet of their German brethren as pupils.—*Home Recorder.*

---

WYETH'S BEEF JUICE is the name of a new Concentrated Extract of Beef just placed on the market by that well-known firm John Wyeth & Bro., of Philadelphia. If it is on a par with the other products of this house, it will only be necessary for them to inform the medical profession that they are ready to supply it. Their advertisement page reads as if they felt sure of their footing in this matter.

---

THE latest strategy of a Paris paper for attracting readers is the engagement of two eminent physicians to attend gratuitously upon its annual subscribers. Recently the manager of the paper gave notice to one of the physicians "not to prescribe for B any more; his subscription has expired." The doctor replied: "So also has B."

---

AN ENGLISH edition of *The Ladies' Home Journal* is to be brought out in London on a scale never before attempted by an American magazine, and Mr. Cyrus H. K. Curtis, proprietor of the *Journal*, and Mr. Edward W. Bok, the editor, sailed for Europe in July to perfect arrangements.

